# The SWISS-MODEL Repository—new features and functionality

**DOI:** 10.1093/nar/gkw1132

**Published:** 2016-11-28

**Authors:** Stefan Bienert, Andrew Waterhouse, Tjaart A. P. de Beer, Gerardo Tauriello, Gabriel Studer, Lorenza Bordoli, Torsten Schwede

**Affiliations:** 1 Biozentrum, University of Basel, Klingelbergstrasse 50–70, CH-4056 Basel, Switzerland; 2 SIB Swiss Institute of Bioinformatics, Biozentrum, University of Basel, Klingelbergstrasse 50–70, CH-4056 Basel, Switzerland

## Abstract

SWISS-MODEL Repository (SMR) is a database of annotated 3D protein structure models generated by the automated SWISS-MODEL homology modeling pipeline. It currently holds >400 000 high quality models covering almost 20% of Swiss-Prot/UniProtKB entries. In this manuscript, we provide an update of features and functionalities which have been implemented recently. We address improvements in target coverage, model quality estimates, functional annotations and improved in-page visualization. We also introduce a new update concept which includes regular updates of an expanded set of core organism models and UniProtKB-based targets, complemented by user-driven on-demand update of individual models. With the new release of the modeling pipeline, SMR has implemented a REST-API and adopted an open licencing model for accessing model coordinates, thus enabling bulk download for groups of targets fostering re-use of models in other contexts. SMR can be accessed at https://swissmodel.expasy.org/repository.

## INTRODUCTION

**Figure 1.**
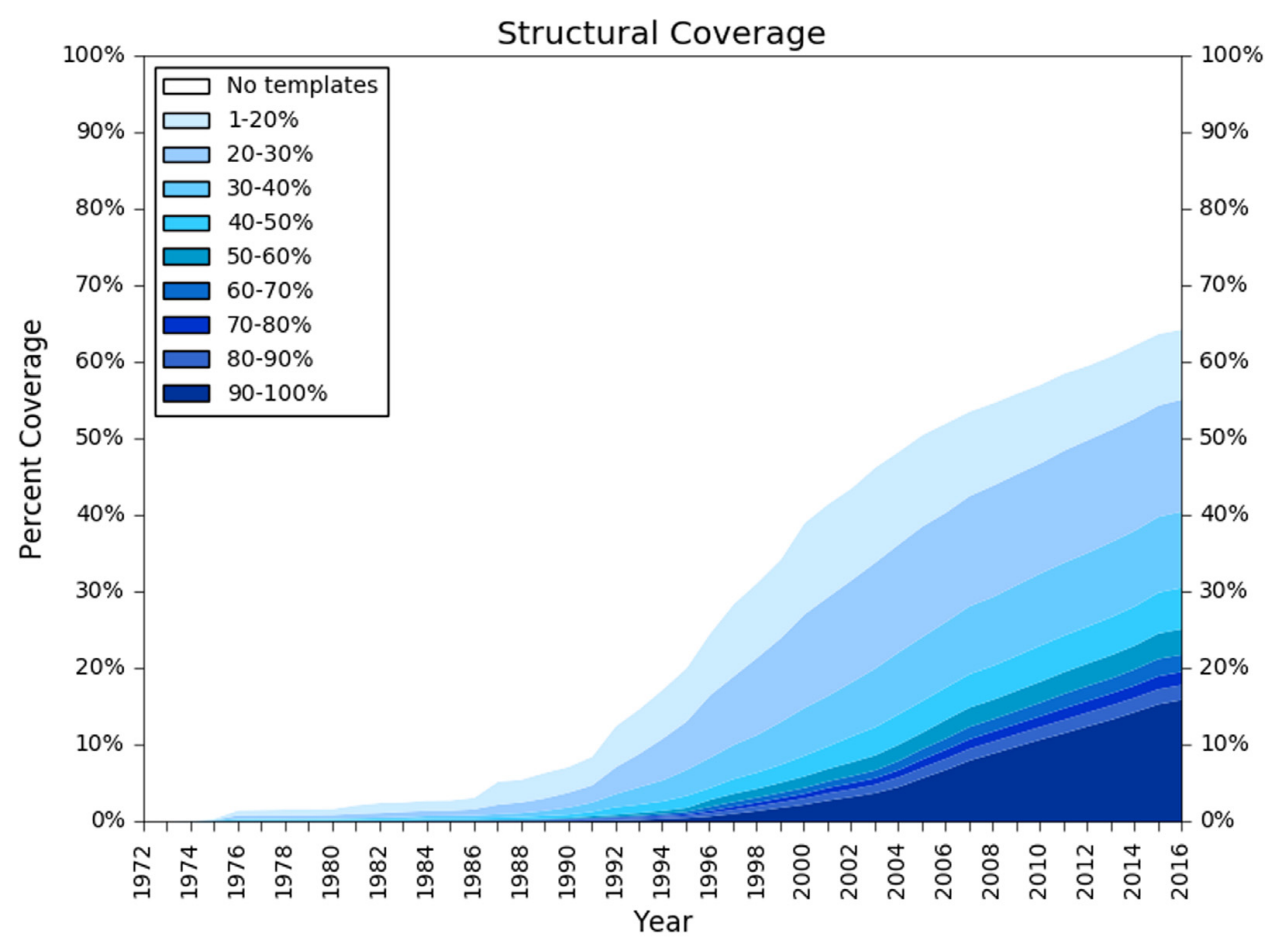
Structural coverage of the human proteome. The plot illustrates the development of structural information for the amino acids of the *Homo sapiens* reference proteome residues (y-axis) over time (adopted from (1)). Profiles were generated for each protein sequence in the reference data set based on the NR20 database and used to search the list of protein sequences in PDB using HHblits (16). For each residue, the highest sequence identity for any alignment to an experimental structure available in a given year was recorded. Different colors in the plot represent the quality of the sequence alignment between the reference proteome sequences (targets) and the sequences of the protein structure database (templates). Alignments with low sequence identity are displayed in light blue, whereas alignments with high sequence identity are depicted in dark blue.

DNA sequencing techniques are generating new sequences at an ever increasing speed. In parallel, the rate at which new protein structures are determined experimentally has increased significantly (1) in recent years, but not at the same speed. As a consequence, only a small fraction of UniProtKB (2) entries have structures deposited in the PDB (3). Computational structural modeling has therefore become a valuable tool for bridging this gap (1). Protein structure homology modeling (aka comparative modeling) is a technique for generating 3D models for proteins, for which experimental structures are not available (targets), based on information derived from homologous proteins with known structure (templates). For example, in 2016 we can identify structural template information (with a sequence identity of at least 30%) for >40% of the human reference proteome (Figure 1). A database of annotated pre-computed homology models allows researchers to explore the protein structure space with little effort and interpret sequence based annotation in the context of the 3D structure. The computational structural biology community has a long tradition of providing protein model databases such as the Genomic Threading Database (4,5), ModBase (6), Genome 3D (7), GPCRdb (8) and SWISS-MODEL Repository (SMR) (9) as well as protein functional annotation databases such as Superfamily (10), CATH (11) and Pfam (12) as valuable services to the life sciences research community.

The specific aim of the SWISS-MODEL Repository is to provide access to an up-to-date collection of high quality annotated 3D protein models and experimental structure information for relevant model organism proteomes and other sequences from UniProtKB. Homology models are generated using the automated SWISS-MODEL (13) homology modeling server pipeline, and experimental structures of proteins are mapped between the PDB and UniProtKB using SIFTS (14).

Here, we report recently implemented features and functionality of SMR. In order to provide an up to date collection of structures and models based on the latest available information, SMR is now updated continuously. Proteins from model organism proteomes are updated on a regular schedule to account for new template information becoming available, while updates for other UniProtKB entries can be initiated interactively by any user from the web interface. New models requested by users are generated by the automated SWISS-MODEL modeling pipeline and released in SMR every 15 min. When selecting templates for modeling, the pipeline aims at optimally covering the length of the target sequence, giving priority to templates that maximize the expected quality of the models and the coverage of the target. In cases where templates mapping to the same sequence segment exhibit significant conformational differences, several models are generated to reflect the structural diversity. All models are assessed and annotated using the QMEAN (15) model quality estimation tool to inform users about the expected local accuracy of the model. Finally, the graphical user interface has been completely redeveloped, implementing novel ways to display structural target coverage and annotations, and the interactive in-page visualization of models.

## THE SWISS-MODEL REPOSITORY

### Improved template library—SMTL

A well curated and up to date template library is crucial for generating high-quality homology models. The SWISS-MODEL Template Library (SMTL) provides sets of atomic coordinates derived from experimental structures in the PDB (3). This set of template structures is curated, e.g. by removing low-quality entries and short peptides, and annotating ligands that are likely to be functionally relevant. Additionally, each entry is annotated with a sequence profile (HMM (16)), predicted secondary structure via SSpro (17) and PSIPRED (18), secondary structure via DSSP (19), predicted solvent accessibility via ACCpro (17), and per residue solvent accessibility via NACCESS (S. Hubbard and J.M. Thornton). The SMTL library of amino acid sequences is searchable by BLAST (20) and HHBlits (16). Structural clustering and superposition of individual chains allows identifying multiple conformations of the same protein. In order to allow modeling of oligomeric structures, SMTL entries are organised as quaternary structure assemblies (based on software and author annotations in PDB). The SMTL is updated on a weekly basis after each new PDB release and currently contains ∼81 000 unique sequences in ∼180 000 assemblies.

### Improvements in the modeling engine—PROMOD3

Our in-house modeling pipeline generates models for both the interactive SWISS-MODEL Server as well as models for the Repository. In brief, the SMTL is searched to identify suitable template structures using BLAST (20) and HHBlits (16) and templates are ranked by a predicted global quality estimate (13). Models are generated based on the highest ranked template, and additional models are progressively generated if new segments of the target sequence can be covered, or if alternative models with significantly different conformations (e.g. open / closed states) can be generated. Oligomeric structures are modeled based on the quaternary structure of the template. At the core of the current pipeline is the new modeling engine ProMod3 that generates the actual model coordinates based on the input alignment (manuscript in preparation). ProMod3 has been implemented based on the OpenStructure (21) library and replaces ProMod-II (22) used previously. Briefly, the main improvements are in loop modeling by making use of an optimized database of loops with each candidate loop being placed using the Cyclic Coordinate Descent (CCD) method (23). The final loop conformation is selected using statistical potentials of mean force. Sidechain modeling is inspired by the work of the Dunbrack lab and uses the 2010 backbone dependent rotamer library (24). After building the model, energy minimization is performed using the OpenMM (25) molecular mechanics library.

### Continuous model database update

SMR aims to provide an up-to-date set of high quality models for a relevant fraction of sequences from the UniProtKB, which currently includes 12 core species (*Homo sapiens, Mus musculus, Caenorhabditis elegans, Escherichia coli K12, Arabidopsis thaliana, Drosophila melanogaster, Saccharomyces cerevisiae, Caulobacter crescentus, Mycobacterium tuberculosis, Pseudomonas aeruginosa, Staphylococcus aureus* and *Plasmodium falciparum*), the Swiss-Prot section of UniProtKB (26), and other protein sequences requested by our users.

In contrast to the previous version of SMR where new models were released several times per year, SMR now features a three-tiered policy for updating: continuously on-demand, weekly, and monthly. Monthly updates are initiated when a new version of UniProtKB is released, i.e. the amino acid sequences of UniProt entries may have changed or the composition of the core species proteomes may have been updated. Every week when the PDB releases new structures, updates of the core species are initiated to ensure that models are based on the latest template information. The latest SIFTS mapping between PDB structures and UniProtKB sequences is imported at this point. For sequences that are not part of the core species data set, we rely on interactive update requests by users. When accessing an entry in SMR for which no models have been built (or the existing models are not based on current template information), two options are presented: a button requesting an automated update of this entry in SMR, or the option for starting an interactive modeling job on SWISS-MODEL Workspace. This allows users to either to schedule an update of the SMR entry in the background, or to interactively explore alternative templates.

In order to provide models on a regular basis we make use of a dedicated Linux cluster of ∼1000 cores to produce on average ∼20 000 models a day. In all cases, the same modeling pipeline is invoked which corresponds to the ‘auto-model’ feature of SWISS-MODEL Workspace. The resulting models are validated (see section on Model Quality below) and if the quality score indicates that the model is of sufficient quality, the new model is inserted into the database. Since SMR aims to provide an up to date collection of high quality models based on latest information, new models build for the same target sequence replace previous versions in the database if they are of higher quality. New models are released typically within 15 min after the model was generated. This continuous release mechanism has the advantage that sequences not part of the core species data set but of interest for users, are regularly updated based on the latest template information and latest version of the SWISS-MODEL modeling pipeline.

### Query and web interface

**Figure 2.**
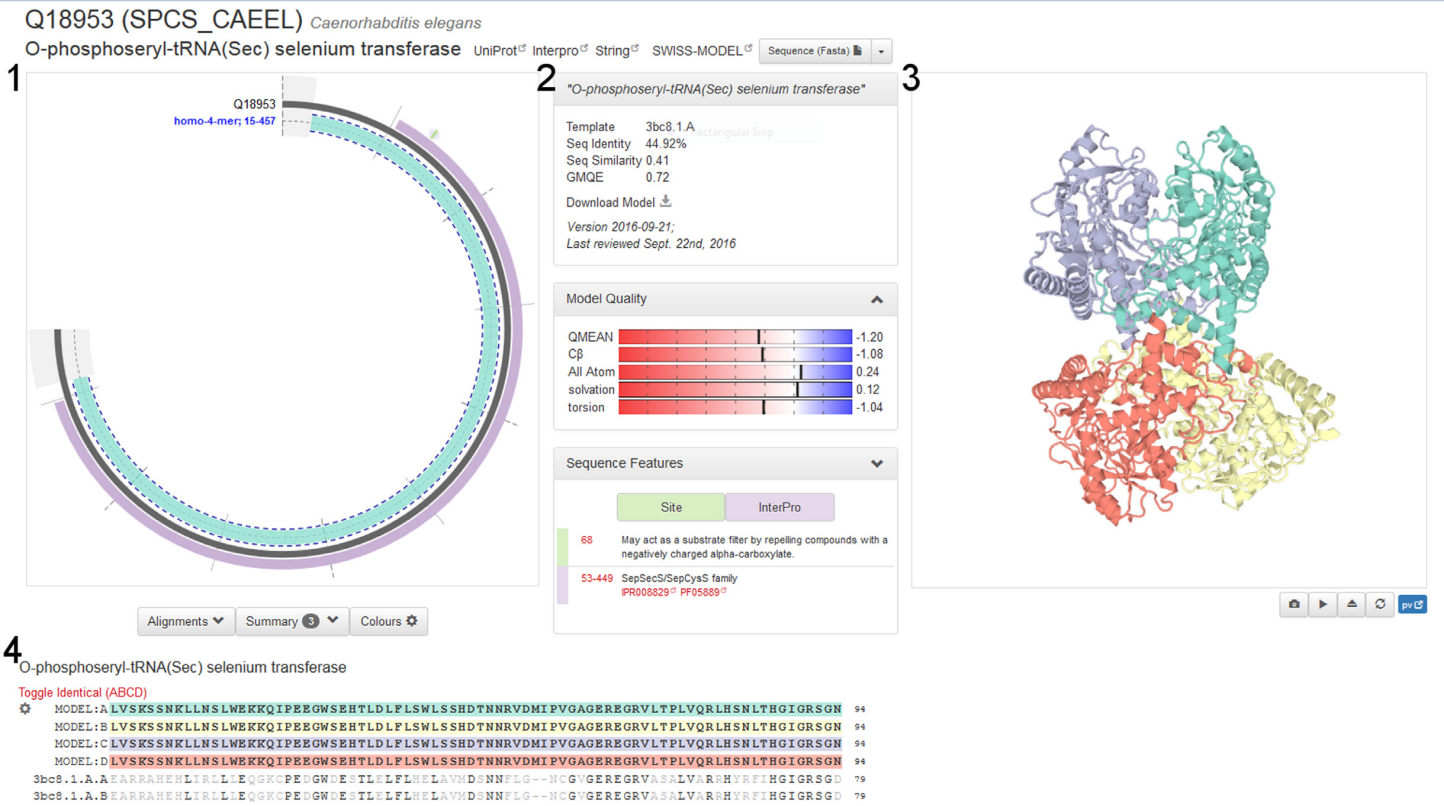
The SWISS-MODEL Repository web page for UniProtKB entry Q18953 (*O*-phosphoseryl-tRNA selenium transferase). The circular representation in section 1 shows the coverage of the target sequence with models and experimental structures. Protein features are annotated on the outside of the arc, in this specific case one InterPro domain and a site annotation. Details about the selected model are shown above the model quality plots in section 2. A Sequence Features drop-down menu reveals a detailed list of feature annotations from UniProt which can be mapped interactively on the model. In section 3, the four chains of the homo-4-mer are highlighted on the 3D model (displayed in PV). The Alignment, Summary, and Colour configuration sections are directly below the protein arc in section 4. The example shown here represents the status of the database at the time of writing and may change over time, e.g. when a new template or better model becomes available.

The SMR web interface provides several entry points for accessing the data. The simplest way to directly access an entry is via its UniProtKB accession code. Alternatively, a free text search on protein names, functional description and organisms allows selecting entries from a list of results matching the search keywords. The entry view page for a specific sequence provides four general sections. The first section (Figure 2, panel 1) is a graphical representation of the coverage by models and experimental structures. The amino acid sequence is indicated as gray arc, with structural coverage indicated at the inside of the arc. A solid segment indicates experimentally determined structures and dashed outlines indicate available homology models. In this view, structures and models are grouped based on their coverage and oligomeric state. Sequence annotation features are displayed on the outside of the arc. FEATURE annotations supplied by UniProtKB include, amongst others, InterPro domains, variants, transmembrane regions, disulphide bonds, nucleotide binding regions, signal peptides and active site residues. Each of these annotations is colour-coded and clickable with a mouse-over showing a brief summary of the specific feature. Additionally, they can also be accessed via the Sequence Features pull-down menu.

The second section (Figure 2, panel 2) provides a summary of the specific SMR entry. Information about the target protein includes the name of the target protein, links to UniProtKB (26), InterPro (27) and STRING (28). For the currently selected model, this section will show which template was used for modeling, provide sequence identity and similarity between target and template, the date on which the model was last updated, an overall model quality estimate (13,15), and provide a link for downloading the specific model coordinates. The model quality plots in this section are providing users with global quality estimates as well as local quality estimates, thus allowing for the identification of unreliable regions (or even residues) of a model. We use an updated version of QMEAN (manuscript in preparation), parameterized for models generated by SWISS-MODEL (13), in order to evaluate the quality of each model and provide three plots. The first plot shows overall quality for each of four measures assessed in QMEAN (Cβ, all-atom, solvation and torsion), the second plot provides a per-residue model confidence estimate, and the third plot shows the QMEAN *Z*-score of the model in comparison with experimental protein structures from the PDB, indicating how similar a given model is in terms of mean force potential to experimental structures (15) of comparable size. In some cases, users may prefer to build models on different template structures than the ones selected automatically, e.g. apo- versus holo-structures or open and closed conformations. For this purpose, a link to SWISS-MODEL workspace is provided, which will start a new interactive modeling session with the respective target sequence preloaded for initiating a new template selection.

The third section (Figure 2, panel 3) features an in-page visualization of the currently selected model/structure, highlighting the functional sequence features selected in the first panel. The graphical view has been implemented using PV (https://biasmv.github.io/pv/), an interactive JavaScript/WebGL based 3D structure viewer. All sections in the SMR page are linked, i.e. selecting a specific feature in the protein sequence representation or sequence alignment will directly highlight this segment in the structure for easy reference and marking a residue in the structure window will highlight the corresponding residues in the sequence alignment.

The fourth section (Figure 2, panel 4) provides details regarding the alignment between the target and the template. The configuration button allows applying various colouring schemes to the sequence and the 3D model shown in PV. Amongst others, the user can colour the alignment and structure by amino acid properties, QMEAN score, by chain or by secondary structure. The configuration button provides export functionalities for the alignment (FASTA or Clustal format as well as a PNG). Below the alignment, a tabular listing of all available experimental structures and models for this protein is provided to allow for an in depth exploration of the available structure information.

### Model quality

SMR aims to provide models that cover as much of the reference sequence as possible while maintaining high quality. The latest SWISS-MODEL pipeline is capable of building homo-oligomeric models, and where appropriate transfers ligand information from the template. The accuracy of the SWISS-MODEL pipeline (13) is continuously benchmarked in comparison with other state-of-the-art methods by the ‘CAMEO’ project (http://cameo3d.org/, (29)) based on the weekly pre-release of PDB sequences.

Each model in SMR is evaluated by QMEAN to provide model quality measures on a per-residue basis as well as a global scale (as discussed above). The graphs provide plots of the estimated local quality of each part of the model as well as how the model compares to other structures in the PDB. In the main overview graph, colours are used to provide quality information at a glance with blue indicating good and red indicating bad quality scores for the specific feature. In order to provide better model quality estimates, we improved the QMEAN (15) algorithm for model quality estimation. It provides both a global quality estimate as well as a per-residue local quality estimate. The original four statistical potential of mean force terms (all-atom interaction, Cβ interaction, solvation, and torsion) have been reformulated and retrained to more specifically meet the needs of ranking higher quality models using a linear combination thereof. To relate the model quality to high resolution X-ray structures of similar size, QMEAN values are expressed as a *Z*-score. Besides the statistical potential of mean force terms, secondary structure and burial status agreement terms have been reformulated to improve local quality prediction (manuscript in preparation). These changes led to an improvement in per-amino acid quality estimation.

### Current repository data content

SMR aims to pre-compute models for some of the most often accessed sequences in UniProtKB (*H. sapiens, M. musculus, C. elegans, E. coli K12, A. thaliana, D. melanogaster, S. cerevisiae, Caulobacter crescentus, M. tuberculosis, P. aeruginosa, Staphylococcus aureus* and *P. falciparum* and the Swiss-Prot section of UniProtKB). For each core species proteome, SMR provides a dedicated summary page with information on the target proteome, statistics on model coverage as well as the evolution of structural coverage (Figure 1) of this proteome over time (e.g. for human http://swissmodel.org/repository/species/9606). The full set of model data for each core species is downloadable from the main entry page as well as the summary page. Models for non-core sequences are added on demand by interactive user request, thereby allowing SMR to dynamically adapt to user needs.

SMR currently contains in excess of 400 000 homology model, complementing 115 663 experimental structures from PDB with mapping to UniProtKB. As of writing, SMR covers ∼20% of Swiss-Prot with high quality models. As new sequences are added dynamically to SMR, coverage will further increase over time. The longest model is 3536 residues (Dynein heavy chain (UniProt: A0A143ZY83) from *P. falciparum*) while an average model size of 232 residues is found in SMR. Table 1 shows the statistics for each of the reference proteomes in the core species.

**Table 1.** Statistics for each of the core species’ canonical sequence sets.

Species	# of sequences in ref. proteome	# of sequences with >1 model	≥ 80%	≥60%	≥40%	≥20%	<20%	No template
*H. sapiens*	21 006	15 195	5010	3299	2673	2648	1965	5411
*M. musculus*	22 274	16 860	6331	3337	2789	2765	1638	5414
*C. elegans*	20 071	10 566	3489	1917	1852	2026	1282	9505
*E. coli K12*	4306	3306	2620	274	211	132	69	1000
*A. thaliana*	27 252	17 132	6544	3335	2772	3004	1477	10 120
*D. melanogaster*	13 704	8502	2956	1488	1309	1525	1224	5202
*S. cerevisiae*	6721	4101	1665	590	550	751	545	2620
*C. crescentus*	3715	2633	1914	303	191	154	71	1082
*M. tuberculosis*	3987	2921	2000	338	275	205	103	1066
*P. aeruginosa*	5550	4270	3271	405	311	199	84	1280
*S. aureus*	2881	1925	1500	170	117	94	44	956
*P. falciparum*	5340	2781	722	352	382	535	790	2559

For each species we show the total number of canonical sequences in the reference proteome (according to UniProtKB), the number of sequences for which we have at least one model, followed by the number of sequences that have models that cover at least 80% (60%, 40%, etc.) of the respective reference sequence.

In order to foster usage of models for different applications in different contexts, we have adopted an open license model for all data provided by SWISS-MODEL Workspace and Repository (Creative Commons CC BY-SA 4.0).

### Cross-links and programmatic access

By providing an up-to-date representation of structural information—both experimental structures and high quality homology models—SMR allows to dynamically map sequence based annotation into a 3D structure context. Cross-links to SMR are currently provided by UniProtKB, InterPro, STRING and PMP Protein Model Portal (29). Annotation from UniProtKB is also used in SMR for visualizing functionally relevant features on models and structures. InterPro supports the functional analysis of protein sequences by classifying them into families and predicting the presence of domains and important sites. The STRING database of functional protein associations allows users explore these interaction networks—bidirectional crosslinking with SMR facilitates the interpretation of interactions from a protein structure perspective. The PMP Protein Model Portal aggregates information from different model providers and allows for a comparative analysis of independently generated models.

Using UniProtKB as reference system for mapping sequence positons to SMR entries greatly facilitates the usage of structure information for developers of other resources. SMR features a RESTful API for programmatically querying the database and downloading models and structures. For example, the API allows querying for structural information available for a defined sequence segment of a specific UniProtKB entry, resulting in a JSON formatted answer specifying all homology models and experimental structures overlapping with the segment, their mapping to the query sequence, and the URL to access the coordinates for each model or structure, respectively. Please note that models in SMR as well as experimental structures retrieved via the API may be oligomers or complexes—and not only single chain models. Documentation and usage examples for the API can be found on the SMR website (https://swissmodel.expasy.org/docs/repository_help).

### Using and publishing homology models

Homology models are based on the available information for a given protein at the time of modeling. When using models for a protein of interest, it is therefore advisable to regularly assess if in the meantime a model of higher quality could be generated due to better templates becoming available. Depending on the evolutionary distance between the target and the available templates, and the structural variability of the protein family of interest, the accuracy of homology models can vary significantly. Ultimately, the requirements of a specific application determine if a model at hand is suitable for the intended purpose (30). Several approaches for model quality estimation have been developed in recent years to assist in determining the expected quality of models (see e.g. (31) for a recent benchmark of various tools).

Models should always be downloaded and stored locally (coordinates as well as meta information such as template alignment etc.) as the corresponding SMR entry might get updated over time. When publishing work that was based on model information, it is good practice to provide access to the models to ensure reproducibility of the work, e.g. by depositing the model in a public open data archive such as Model Archive (http://www.modelarchive.org/) and providing the stable reference code to the model in the manuscript (30,32).

### Technical implementation

The current version of SMR was developed using the Django framework (https://www.djangoproject.com) with a redundant MongoDB (https://www.mongodb.com) implementation as the database. All graphical aspects of the website are implemented using jQuery (https://jquery.com), RaphaelJS (https://github.com/DmitryBaranovskiy/raphael) and PV (https://biasmv.github.io/pv/).

## FUTURE DIRECTIONS

In the future SMR will focus on increasing the impact of models for practical applications by improving coverage and completeness. We plan to improve the modeling pipeline with respect to modeling quaternary structures for more distant homologs, extending it to hetero-oligomeric complexes, and aim to broaden the spectrum of biologically relevant ligands and co-factors in the predicted structures.

In terms of user interface, we envisage to extend the current API by a graphical JS based widget that allows developers of other websites to map their information in a 3D structure context. Future releases of SMR will be based on the mmCIF model dictionary extension currently being developed in the context of the wwPDB task force on hybrid modeling (33).
